# Porous 3D Prussian blue/cellulose aerogel as a decorporation agent for removal of ingested cesium from the gastrointestinal tract

**DOI:** 10.1038/s41598-018-22715-w

**Published:** 2018-03-14

**Authors:** Ilsong Lee, Sung-Hyun Kim, Muruganantham Rethinasabapathy, Yuvaraj Haldorai, Go-Woon Lee, Sang Rak Choe, Sung-Chan Jang, Sung-Min Kang, Young-Kyu Han, Changhyun Roh, Wan-Seob Cho, Yun Suk Huh

**Affiliations:** 1 Department of Biological Engineering, Biohybrid Systems Research Center (BSRC), Inha University, 100, Inha-ro, Incheon, 22212 Republic of Korea; 2 Biotechnology Research Division, Advanced Radiation Technology Institute (ARTI), Korea Atomic Energy Research Institute (KAERI), 29, Geumgu-gil, Jeongeup-si, Jeonbuk, 56212 Republic of Korea; 3 Laboratory of Toxicology, Department of Medicinal Biotechnology, College of Health Sciences, Dong-A University, 37, Nakdong-daero, 550 beon-gil, Busan, 49315 Republic of Korea; 4 Department of Energy and Materials Engineering, Dongguk University-Seoul, 30 Pildong-ro 1-gil, Seoul, 04620 Republic of Korea; 5 R&D Platform Center, Korea Institute of Energy Research (KIER), 152, Gajeong-ro, Deajeon, 34129 Republic of Korea; 6 Radiation Biotechnology and Applied Radioisotope Science, University of Science and Technology (UST), 217, Gajeong-ro, Daejeon, 34113 Republic of Korea; 7 Present Address: Department of Nanoscience and Technology, Bharathiar University, Coimbatore, 641 046 Tamilnadu India

## Abstract

In the present study, we successfully synthesized a porous three-dimensional Prussian blue-cellulose aerogel (PB-CA) composite and used it as a decorporation agent for the selective removal of ingested cesium ions (Cs^+^) from the gastrointestinal (GI) tract. The safety of the PB-CA composite was evaluated through an *in vitro* cytotoxicity study using macrophage-like THP-1 cells and Caco-2 intestinal epithelial cells. The results revealed that the PB-CA composite was not cytotoxic. An adsorption study to examine the efficiency of the decorporation agent was conducted using a simulated intestinal fluid (SIF). The adsorption isotherm was fitted to the Langmuir model with a maximum Cs^+^ adsorption capacity of 13.70 mg/g in SIF that followed pseudo-second-order kinetics. The PB-CA composite showed excellent stability in SIF with a maximum Cs^+^ removal efficiency of 99.43%. The promising safety toxicology profile, remarkable Cs^+^ adsorption efficacy, and excellent stability of the composite demonstrated its great potential for use as an orally administered drug for the decorporation of Cs^+^ from the GI tract.

## Introduction

A considerable amount of radionuclides have been released into the environment owing to the use of nuclear explosive devices or radiological dirty bombs and enter the human body via inhalation, ingestion, and wound contamination^1–6^. All radionuclides, whether primarily ingested from contaminated food and water or secondarily ingested via the respiratory tract, will enter the systemic circulation^7,8^ and may pose significant health risks to the exposed individuals^9^ depending on the dose of the radioactive contaminant and the biological status of the subject, such as age and health. The gastrointestinal (GI) tract is a critical target organ for many insoluble radioactive contaminants owing to contaminants traveling the length of the tract unabsorbed and the excretion via hepatobiliary clearance. Thus, it is important to develop a safe and effective procedure for the removal of radionuclides from the body after contamination^10^.

Radioactive cesium (^137^Cs) is the most harmful naturally occurring radionuclide, with a long half-life (30.17 years) and high water solubility and mobility, which readily enters the animal and human food chains through the consumption of contaminated water, plants, meat, fish, and milk^11,12^. Moreover, Cs in animals and humans is processed pharmacokinetically in the same way as sodium (Na) and potassium (K) owing to its chemical analogy with those elements^13,14^. Approximately 10% of Cs is eliminated rapidly with a biological half-life of 2 days, 90% is eliminated gradually with a biological half-life of 110 days, and less than 1% remains with a longer biological half-life of approximately 500 days^15^. Decorporation agents enhance the elimination or excretion of absorbed radioactive contaminants, are associated with the absorption of ^137^Cs from the GI tract into the systemic circulation, and improve elimination after absorption; therefore, they are of great use for the minimization of the absorbed radiation dose when people are exposed to these radionuclides^4,16^. Owing to the similar biological nature of Cs and Na/K, decorporation agents should have a high selectivity for Cs to avoid electrolytic imbalances caused by the elimination of Na and K from the GI tract^1,17–19^.

Prussian blue (PB; trade name Radiogardase®) is the only drug that is currently approved by the U.S. Food and Drug Administration (FDA) and European Medicines Agency for the decorporation of internal Cs contamination^20,21^. The side effects of PB include constipation and undefined gastric distress^22^ may increase radiation exposure by increasing the transit time of ^137^Cs. In addition, recent developments in nanoparticulate PB have exposed some latent problems, such as absorption through intestinal epithelial cells, agglomeration in neutral buffered conditions, and binding to other elements (*e.g*., K), which result in electrolyte imbalance. Therefore, the practical application of these decorporation agents has limitations from the perspectives of safety and efficacy.

Highly efficient clinically acceptable decorporation agents should be developed through the elimination of the safety issues related to the existing decorporation agents. In a previous study, two types of PB composite, based on nanoporous silica or carbon sponge, were suggested for internal Cs removal^23,24^. Currently, intensive research has focused on the viability of natural polymers, such as alginate, chitosan, collagen, starch, and cellulose, for use in a range of biomedical applications. Among these polymers, cellulose has emerged as an increasingly attractive compound owing to its excellent physical stability and biocompatibility^25^. In addition, the strongly interacting hydroxyl functional groups present in cellulose confer a strong affinity for self-association and form an extended network through inter- and intra-molecular hydrogen bonds. In particular, the cellulose materials have an ultrafine nanofibrous network structure with high strength and are not digested in the human GI tract. Cellulose is approved and regarded as safe by the U. S. FDA^19,26–29^.

In this study, we synthesized an orally applicable, 3D, porous PB-CA composite and examined the decorporation of cesium ion (Cs^+^) from the GI tract. The cytotoxicity and cell viability were tested using macrophage-like THP-1 cells and Caco-2 intestinal epithelial cells. In order to investigate the efficiency of the decorporation agent, adsorption isotherm studies, kinetic analyses, and stability tests were conducted in simulated gastric fluid (SGF) and simulated intestinal fluid (SIF) to mimic GI fluid, and gamma ray irradiated condition.

## Results and Discussion

### Synthesis, and structural, surface, and morphological studies

**Figure 1:**
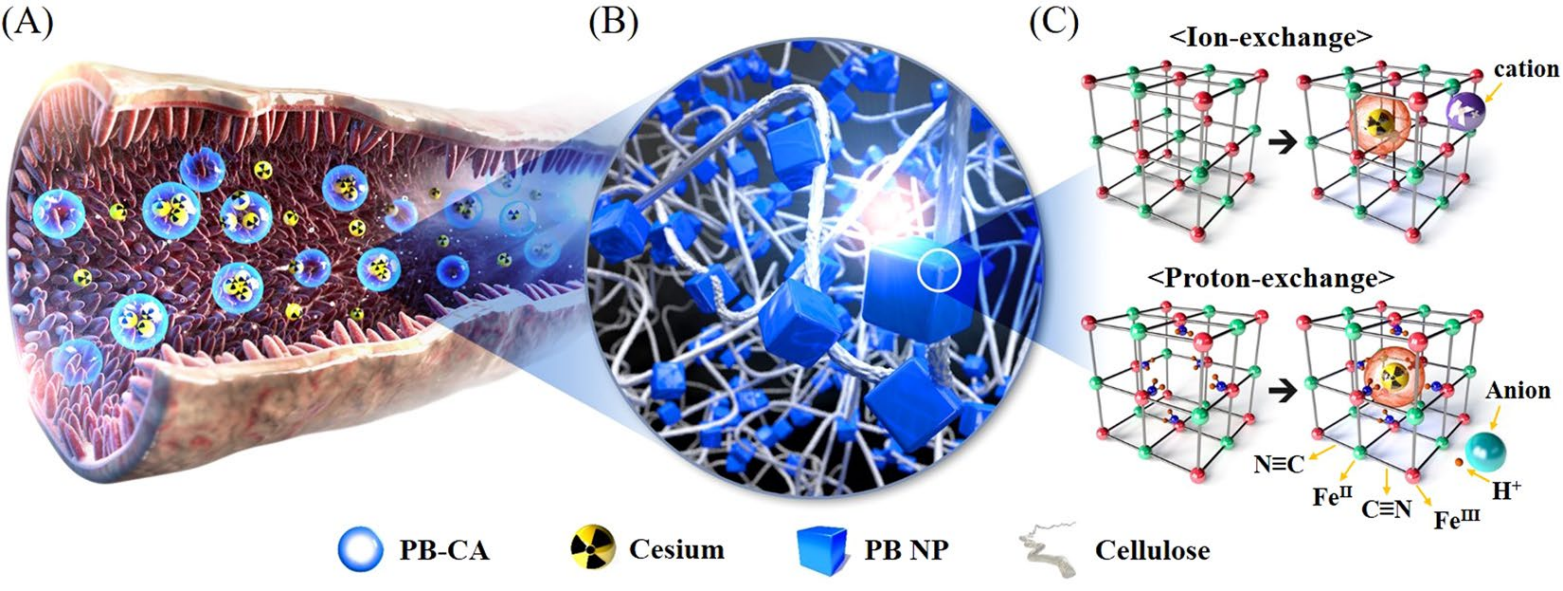
(**A**) Schematic diagram of the action of PB-CA in the gastrointestinal tract. (**B**) Illustrative morphology of PB-CA composite. (**C**) Cesium adsorption mechanism of PB.

A schematic illustration of the GI tract, in which decorporation of Cs^+^ takes place by the PB-CA composite, is shown in Fig. 1A. The tight caging of Prussian blue in cellulose matrix is schematically represented in Figure B. While considering the mechanism of Cs adsorption by PB, both ion- and proton-exchange takes place together^30^. In ion-exchange process, Cs is adsorbed by physical adsorption in the regular lattice spaces of PB through cation exchange (Fig. 1C, upper panel). Whereas, the chemical adsorption with proton-exchange is the major Cs adsorption mechanism in which Cs is adsorbed by the hydrophilic defect site of PB with proton elimination from the coordinated water (Fig. 1C, lower panel). The decorporation agents to remove Cs are normally orally administered and are expected to be absorbed in the intestinal sections of the GI tract^20,31^.

**Figure 2:**
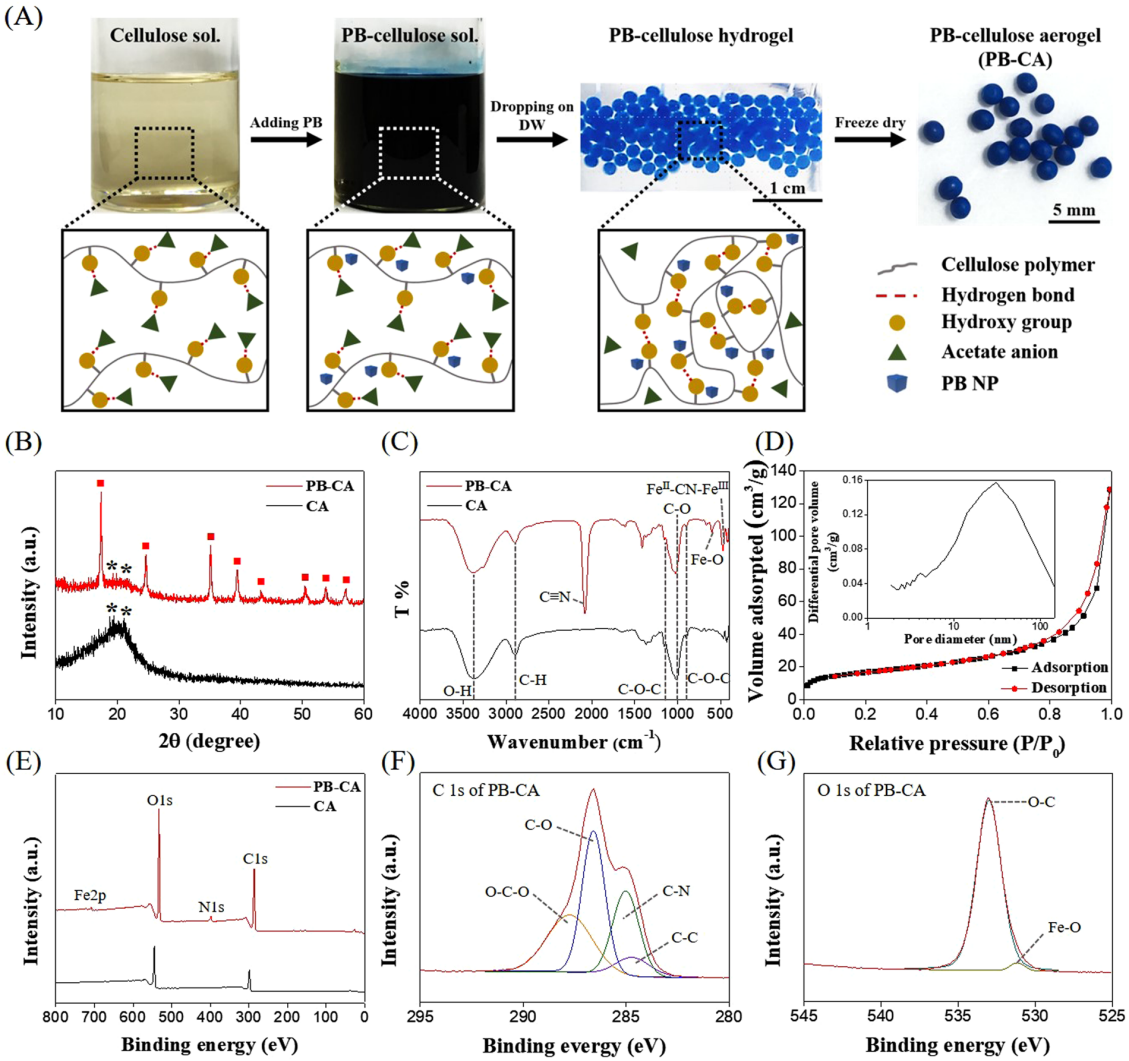
Fabrication and characterization of PB-CA. (**A**) Schematic diagram of the fabrication of PB-CA composite, (**B**) XRD patterns of CA and PB-CA composite, (**C**) FT-IR spectra of CA and PB-CA composite, (**D**) BET isotherm of PB-CA composite (inset: pore size distribution of PB-CA composite), (**E**) XPS survey spectra of CA and PB-CA composite, (**F**) C 1s spectra of PB-CA composite, and (**G**) O 1s spectra of PB-CA composite.

A schematic diagram of the fabrication of the PB-CA composite is shown in Fig. 2A. During the synthesis process, the acetate ions present in tetrabutylammonium acetate (TBAA) of TBAA/DMSO (tetrabutylammonium acetate/dimethyl sulfoxide) (solvent to dissolve cellulose) block the hydroxyl groups of cellulose through the formation of new hydrogen bonds^32^ that disrupt the intra- and inter-molecular hydrogen bonding^33^ to form cellulose solution and then PB-cellulose solution by the addition of PB. The PB-cellulose solution was then added dropwise to DW in room temperature to form PB-cellulose hydrogel with immediate gelation which may be attributed to the diffusion of acetate ions from the hydroxyl group of cellulose to DW. Finally, PB-cellulose aerogel (PB-CA) was obtained by freeze drying.

Figure 2B shows the XRD patterns of CA and the PB-CA composite. The XRD pattern of CA showed two weak peaks at 19.6° and 21.6°, which corresponded to the (101) and (002) reflections, respectively^25,34–36^. The PB-CA composite exhibited intense characteristic peaks of PB at 2θ values of 17.5°, 24.9°, 35.5°, 39.5°, 43.5°, 50.7°, 54.0°, and 57.1°, which corresponded to the (200), (220), (400), (420), (422), (440), (600), and (620) planes, respectively, along with the less intense characteristic peaks of cellulose^37–39^, which confirmed the formation of the composite.

Figure 2C compares the FT-IR spectra of CA and the PB-CA composite. The FT-IR spectrum of CA showed characteristic bands of cellulose at 3430, 2901, 1163, 1024, and 892 cm^−1^ corresponding to O-H, C-H, C-O-C of the glycosidic bond, C-O stretching, and C-H deformation, respectively^26,40,41^. The same set of bands were observed for the PB-CA composite. The Fe-O band at 601 cm^−1^ due to the interaction between the hydroxyl groups of cellulose and the ferric ions of PB^25,42,43^ (Figure S1A) confirms the enhanced interaction with cellulose and PB^44^. As a reference, the FT-IR spectrum of pure PB and cellulose is shown in Figure S1B. The bands at 2080 and 468 cm^−1^ corresponded to the C=N stretching and Fe^II^-CN-Fe^III^ formation^38,42,45^, respectively.

The Brunauer-Emmett-Teller (BET) specific surface area of the PB-CA composite and CA were 58.31 (Fig. 2D) and 43 m^2^ g^−1^ (Figure S1C), respectively. The addition of PB NPs onto CA increased the volume of porous CA with increased surface area of the composite which facilitated the rapid diffusion of Cs^+^ into the composite, allowing virtually all of the PB sites to bind Cs^+^, and resulted in a larger absorption capacity and faster kinetics. The PB-CA composite exhibited mesopores (2–50 nm) and macropores (>50 nm) (Fig. 2D, inset) related to the Type IV and V adsorption isotherm of IUPAC classification^46^.

The XPS survey scan of the PB-CA composite (Fig. 2E) showed C1s, N1s, O1s, and Fe2p peaks with binding energies of 286.56, 397.72, 533.0, and 708.46 eV, respectively. The C1s spectrum (Fig. 2F) of the composite was deconvoluted into four constituents: C-C (284.70 eV), C-N (285.02 eV), C-O (286.59 eV), and O-C-O (287.74 eV). The C-N peak demonstrated the existence of PB NPs in the composite, which was absent in the spectrum of CA (Figure S1D). The O1s spectrum (Fig. 2G) exhibited two peaks at 286.59 eV and 531.18 eV, corresponding to C–O^35^ and Fe–O^44^, respectively. The spectrum clearly indicated a chemical bond between PB and cellulose (Figure S1A) based on the presence of the Fe-O peak that was absent in the deconvoluted O 1 s spectrum of CA^26,47^ (Figure S1E). The Fe 2p spectrum of the PB-CA composite was deconvoluted into three peaks (Figure S1F) with binding energies of 708.48 eV, 712.18 eV and 721.68 eV corresponded to the ferrous ions of ferrocyanide, the high spin (Fe 2p_3/2_) and low spin (Fe 2p_1/2_) states of ferric ions^48,49^, respectively.

**Figure 3:**
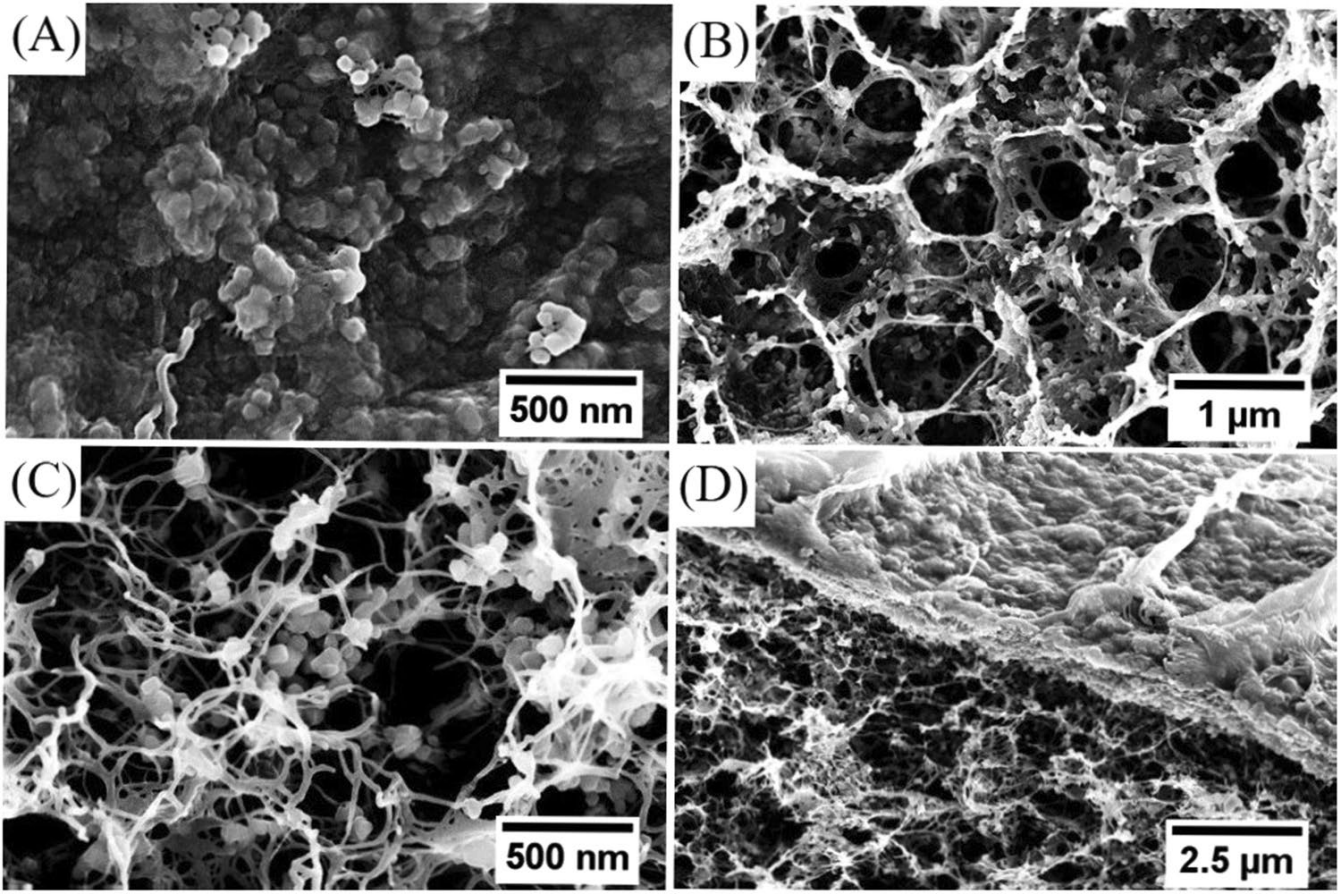
SEM images of the PB-CA composite. (**A**) Surface morphology, (**B**,**C**) inner morphology at different magnifications, and (**D**) cross section morphology.

Figure 3 shows the SEM images of the PB-CA composite. As shown in Fig. 3A and Figure S2A, the composite was highly dense and the surface was rough. This surface roughness may result from the tightly packed PB NPs inside the cellulose matrix. Figure 3B and C shows the 3D porous network structure of PB-CA composite, in which cubic PB NPs with an average particle size of 50 nm were uniformly dispersed with dense packing. In addition, the porous fibrillar network structure of cellulose was retained and stable in the composite, even when loaded with PB. A cross-sectional SEM image of the composite is also shown in Fig. 3D. For reference, the SEM images of the surface and inner structure of the CA are displayed in Figure S2B and C.

### Cytotoxicity of PB and the PB-CA composite

**Figure 4:**
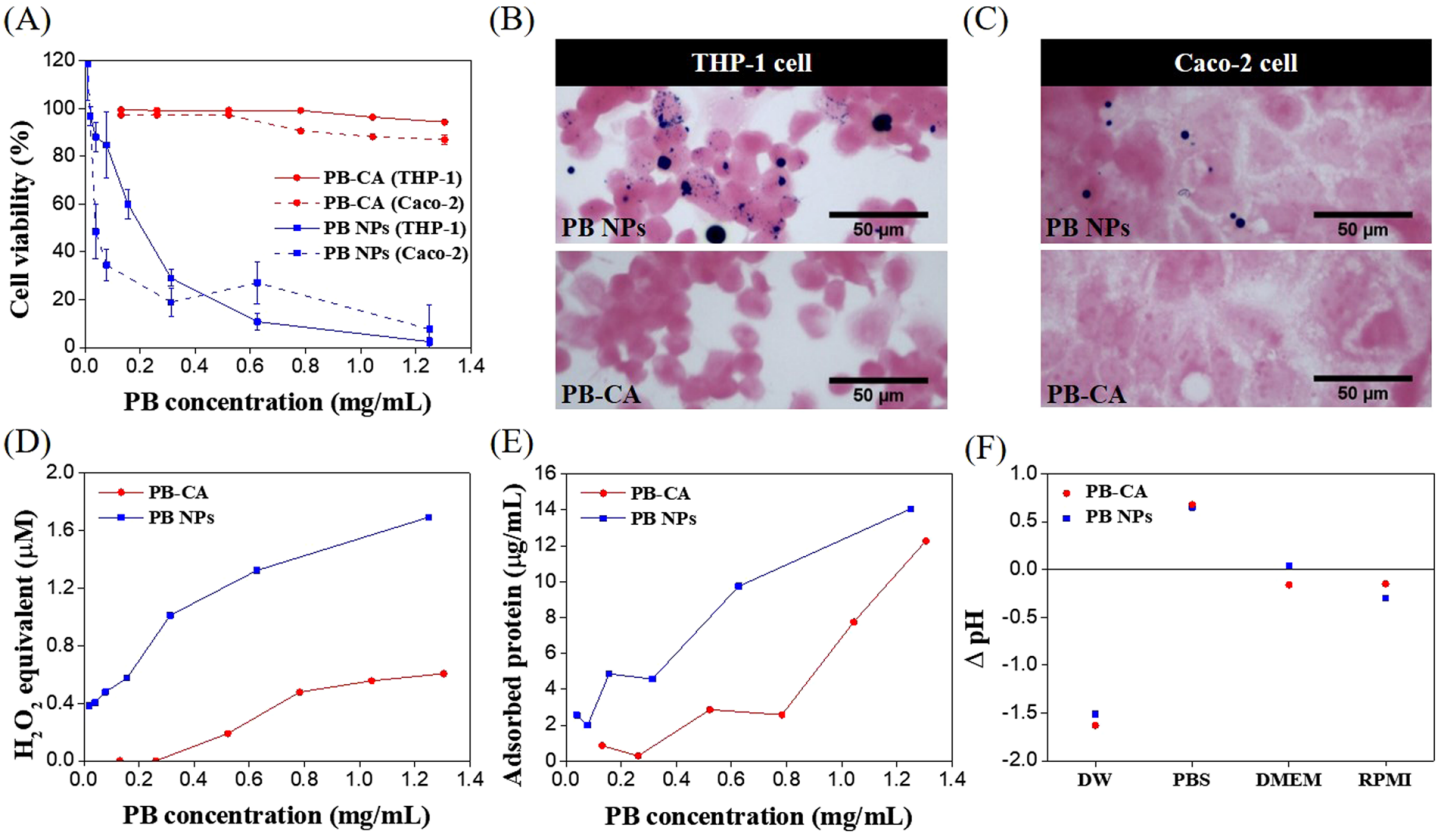
Cell viability analysis of PB and PB-CA (direct and indirect mechanisms). (**A**) Cytotoxicity of PB and PB-CA in macrophage-like THP-1 cells (solid line) and Caco-2 intestinal epithelial cells (dashed line). (**B**) Optical images of macrophage-like THP-1 cells treated by PB NPs (upper panel) and PB-CA (lower panel). (**C**) Optical images of Caco-2 intestinal epithelial cells treated by PB NPs (upper panel) and PB-CA (lower panel). (**D**) Potential of ROS generation of PB and PB-CA measured by DCFH-DA assay. (**E**) Protein corona binding affinity assay of PB and PB-CA (the data are expressed as adsorbed protein levels). (**F**) The change of pH before and after incubation of PB and PB-CA in various media.

To evaluate cell viability, we selected macrophage-like THP-1 cells that represent the cells of the innate immune system against the invasion of foreign materials and Caco-2 intestinal epithelial cells, which are the major type of cells that interact with swallowed decorporation agents. In macrophage-like THP-1 cells, PB was strongly cytotoxic and the effective concentration required to effect a 50% reduction in growth (EC_50_) was approximately 205 μg/mL; however, the PB-CA composite did not show any cytotoxicity within the tested dosage range (Fig. 4A, solid line). In Caco-2 intestinal epithelial cells, PB showed strong cytotoxicity with an EC_50_ of approximately 96 μg/mL, whereas the PB-CA composite was not cytotoxic at the tested dosage (Fig. 4A, dashed line).

The differences in cytotoxicity of the PB NPs may result from the differences in the efficiency of their cellular uptake. In macrophage-like THP-1 cells, some PB NPs were endocytosed, but most were not; however, the PB-CA composite was not endocytosed at all, which may be attributable to the large size of the PB-CA composite (Fig. 4B). In Caco-2 intestinal epithelial cells, neither PB nor the PB-CA composite was endocytosed (Fig. 4C). The time-course observations of the agglomeration effects of PB showed that PB NPs were well dispersed in DW, but severe agglomeration was evident in PBS and cell culture media (DMEM and RPMI-1640) (Figure S3). The PB NPs agglomerated immediately after dispersion and this continued for 12 h. The high tendency for agglomeration could explain the minimal uptake of PB in both cell types. Oxidative stress is often considered a key factor for cell damage induced by PB NPs. The ROS level measured by the cell-free DCFH-DA assay showed that PB had a more marked ROS burst than that of the PB-CA composite and the dose-response curve of ROS of PB was consistent with its cytotoxicity pattern (Fig. 4D).

In biological environments such as cell culture media and body fluids, the surface of NPs will be shielded by the formation of a “protein corona” that can alter the surface charge and active residues on the particles, which can result in behavioral changes in biological media^50^. In the protein binding assay, PB showed a higher adsorption capacity than that of the PB-CA composite because of its high surface area and high binding affinity towards the protein (Fig. 4E). The acidification of the cell culture medium by the release of protons (H^+^) from PB or the PB-CA composite can be considered as an indirect cytotoxicity mechanism. The pH of the culture medium was measured before and after the addition of PB or PB-CA. Briefly, PB or the PB-CA composite was dispersed in DMEM medium at 800 μg/mL. The suspensions were incubated for 24 h under the same conditions as the cell culture study and the pH was measured. The pH changes of PB and the PB-CA composite before and after incubation for 24 h with different cell culture media are shown in Fig. 4F. Small changes were observed in pH, which suggested that the protons released upon the binding of PB with the salts of the culture media did not cause any significant changes in cytotoxicity.

### Physicochemical stability of PB-CA composite

**Figure 5:**
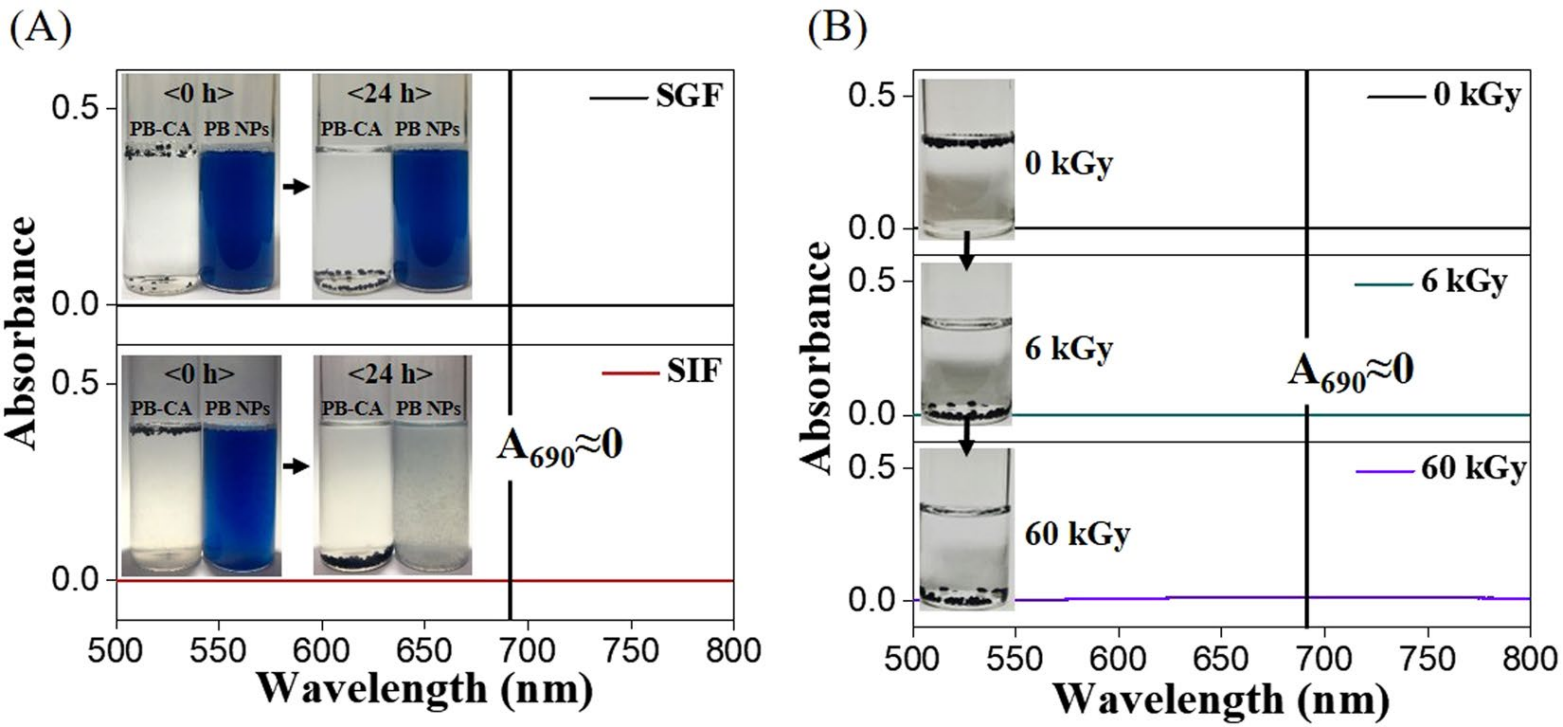
Adsorption stability test of PB-CA. (**A**) UV spectra of PB-CA treated in SGF (upper panel) and SIF (lower panel) for 24 h (the insets present optical microscopy images to show the stability behavior of PB NPs and PB-CA in SGF (upper panel) and SIF (lower panel) treated for 24 h). (**B**) UV spectra of PB-CA after gamma ray irradiated at 0 kGy (upper panel), 6 kGy (middle panel), and 60 kGy (lower panel) (the insets represent optical microscopy images to show the behavior of PB-CA after gamma ray irradiation at 0 kGy (upper panel), 6 kGy (middle panel), and 60 kGy (lower panel)).

An understanding of the stability and PB-release of the PB-CA composite is important to determine the suitability of the PB-CA composite as an orally administered decorporation agent for the removal of Cs from the GI tract. A number of physiochemical factors can play important roles in the stability and release of PB from the composite. The potential effects of digestive fluid, which contains various enzymes, salts, and pH conditions, and gamma-rays emitted from radioactive Cs are of significant concern. The stability and PB release tests were conducted by the dispersion of the PB-CA composite in SGF and SIF (to simulate real conditions) for 24 h and the solutions were analyzed by UV-vis spectroscopy. As shown in Fig. 5A, the composite retained its structure without the release of PB, even after treatment for 24 h, and the sample remained transparent with no turbidity or phase separation. However, the PB NPs were aggregated and deposited in SGF and changed the color of SIF (Fig. 5A, insets). The absorption band at 690 nm that corresponded to PB was absent in the UV-vis spectrum, which confirmed that the PB NPs were not released from the PB-CA composite. A similar experiment was performed to evaluate the influence of gamma-rays on the stability of the PB-CA composite in SGF and SIF by irradiation at different dosages (0, 6, and 60 kGy). As shown in Fig. 5B, the PB-CA composite was stable in both fluids and no decomposition was observed, even at the 60 kGy dose. The absence of the PB absorbance peak at approximately 690 nm in the UV-vis spectra confirmed that the PB NPs were not released from the PB-CA composite. This *in vitro* data indicated that the composite was stable and unaffected by gamma radiation, gastric fluid, or intestinal fluid and suggested that the PB-CA composite would outperform PB in terms of stability.

### Adsorption isotherms and kinetic studies

The equilibrium adsorption isotherm process on the surface of the adsorbent was described by Langmuir and Freundlich adsorption isotherm models^51,52^. The classical Langmuir isotherm model refers to homogeneous monolayer adsorption (the adsorbed layer is one molecule thick), in which adsorption can only occur at a finite (fixed) number of identical and equivalent definitively localized sites, with no lateral interactions between the adsorbed molecules, even on adjacent sites^53,54^. The linear and nonlinear forms of the Langmuir isotherm^39,43^ are written as:

1 $$\frac{{C}_{e}}{{q}_{e}}=\frac{1}{{K}_{L}}+\frac{{C}_{e}}{{q}_{max}}$$

2 $${q}_{e}={q}_{max}\frac{{K}_{L}{C}_{e}}{1+{K}_{L}{C}_{e}}$$

where *q*_*e*_ is the equilibrium adsorption capacity (mg/g) and *q*_*max*_ is the monolayer maximum adsorption capacity (mg/g), respectively; *C*_*e*_ is the equilibrium Cs^+^ concentration of the aqueous phase, and *K*_*L*_ is affinity coefficient between the adsorbent and adsorbate.

The Freundlich adsorption isotherm model is used to describe the adsorption characteristics for a heterogeneous surface and can be applied to the multilayer adsorption of an adsorbate over an adsorbent surface. The Freundlich isotherm is expressed by the equations:

3 $$ln\,{q}_{e}=\,ln\,{K}_{F}+\frac{\mathrm{ln}\,{C}_{e}}{n}$$

4 $${q}_{e}={K}_{F}{{C}_{e}}^{\frac{1}{n}}$$

where *K*_*F*_ is the measured adsorption capacity of the adsorbent and 1/*n* is the adsorption intensity.

**Figure 6:**
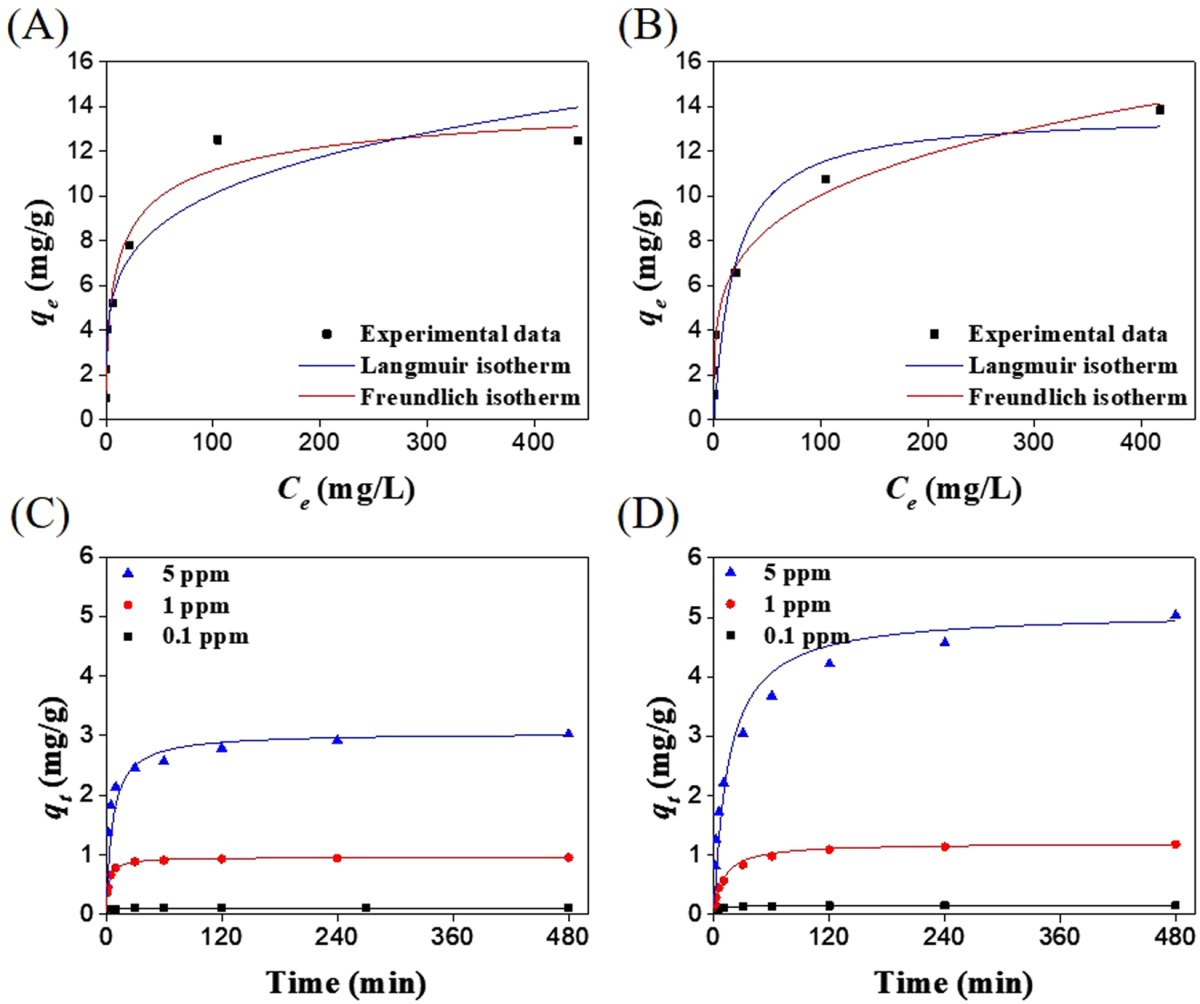
Cesium adsorption isotherm and kinetics studies of PB-CA. (**A**) Cesium adsorption isotherms fitted with Langmuir and Freundlich models in DW. (**B**) Cesium adsorption isotherms fitted with Langmuir and Freundlich models in SIF. (**C**) Cesium adsorption kinetics of PB-CA in DW fitted with pseudo-second order kinetics model. (**D**) Cesium adsorption kinetics of PB-CA in SIF fitted with pseudo-second order kinetics model.

The Cs^+^ adsorption experiments were performed in DW (Fig. 6A) and SIF (Fig. 6B) and the data were fitted to the Langmuir and Freundlich isotherms. The linear regression correlation (*R*^2^) and Cs^+^ adsorption parameters are listed in Table 1. The adsorption isotherm agreed with the Langmuir model with an excellent fit, which was indicative of the monolayer adsorption of Cs^+^. The maximum Cs^+^ adsorption capacities of the PB-CA composite in DW and SIF were 15.38 and 13.70 mg/g, respectively. Compared with SIF, the PB-CA composite showed higher Cs^+^ adsorption capacity in DW. The lower Cs^+^ adsorption capacity of SIF may be attributed to the presence of a large concentration (approximately 2000 ppm) of competitive K^+^ ions that come into contact with the active adsorption sites of the composite^55^. It has been observed experimentally that with the addition of an excess 10% by weight of the adsorbent, the adsorption capacity of PB-CA in SIF was equivalent to that in DW. The Freundlich factor *n* represents the heterogeneity factor and the *n* value between 1 and 10 determines the favorability of the binding affinity between the adsorbate and adsorbent; a higher *n* value represents stronger binding. In the present study, the calculated values of *n* in DW and SIF were 4.55 and 4.17, respectively, which indicated the physical adsorption of Cs^+^ onto the PB-CA composite.

**Table 1:** Cesium adsorption parameters of the Langmuir and Freundlich models of PB-CA in DW and SIF.

	Langmuir model			Freundlich model		
	*K*_*L*_ [L/mg]	*q*_*max*_ [mg/g]	*R* ^2^	*K*_*F*_ [L/mg]	*n*	*R* ^2^
DW	0.23	15.38	0.9515	3.67	4.55	0.8969
SIF	0.05	13.70	0.8468	3.32	4.17	0.9888

The adsorption kinetics were investigated by pseudo-first- and pseudo-second-order kinetic models to assess the rate of Cs^+^ adsorption and to understand the adsorption process that controlled the reaction mechanism^56,57^. The Cs^+^ adsorption kinetics of the PB-CA composite in DW and SIF are shown in Fig. 6C and D. The pseudo first-order rate equation is given as:

5 $$\frac{d{q}_{t}}{dt}={k}_{1}({q}_{e}-{q}_{t})$$

where *q*_*e*_ and *q*_*t*_ are the adsorption capacities (mg/g) at equilibrium and at time *t*, respectively, and *k*_1_ is the pseudo-first-order rate constant (min^−1^). Upon integration and application of the boundary conditions, *t* = 0 to *t* = *t* and *q*_*t*_ = 0 to *q*_*t*_ = *q*_*e*_, a simple linear form of the rate equation was obtained:

6 $$ln({q}_{e}-{q}_{t})=ln{q}_{e}-{k}_{1}t$$

In Figure S4A and B, the plots of *ln(q*_*e*_ − *q*_*t*_) versus *t* are shown and Table S1 lists the kinetic parameters.

The pseudo-second-order kinetic model assumes that the reaction kinetics were influenced not only by Cs^+^ concentration, but also by the active sites on the adsorbent. The pseudo-second-order model is represented by the following equations:

7 $$\frac{d{q}_{t}}{dt}={k}_{2}{({q}_{e}-{q}_{t})}^{2}$$

8 $$\frac{t}{{q}_{t}}=\frac{1}{{k}_{2}{{q}_{e}}^{2}}+\frac{1}{{q}_{e}}t$$

The second-order rate constant (*k*_2_) and equilibrium adsorption capacities (*q*_*e*_) were calculated from the linear plot of *t/q*_*t*_ vs *t* using equation (8) (Figure S4C and D). Compared with the first-order model, the second-order model had a higher correlation coefficient, which implied that the Cs^+^ was chemisorbed and the adsorption rate of the composite depended on the active sites rather than the concentration of Cs^+^ in the solution.

**Figure 7:**
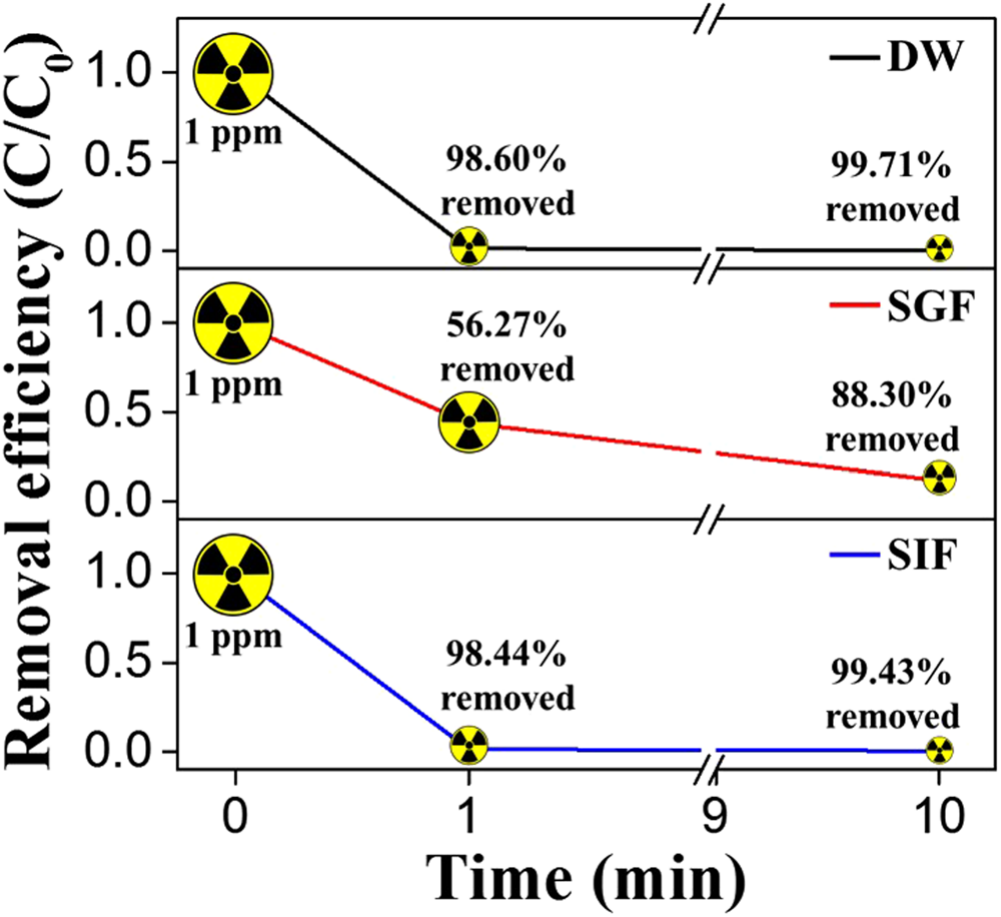
Cesium removal efficiency in DW, SGF, and SIF after 1 and 10 min (10 mL of cesium solution with 50 mg of PB-CA).

The kinetic studies showed that the equilibrium Cs^+^ adsorption capacity of the composite was higher in SIF than DW, even though the rate of reaction was faster in DW (Table S1). The difference between the equilibrium adsorption capacities of the composite in DW and SIF can be explained by the differences in the pH value of the aqueous solution, which is a significant parameter in controlling the adsorption process. The PB in the PB-CA composite possesses a cubic lattice structure with Fe(II) and Fe(III) occupying the corners of the cube and the cyanide group positioned on the sides along with the presence of 14–16 coordinated water molecules. Normally, Cs^+^ are completely trapped by chemical adsorption through hydrophilic lattice defect sites of PB and the proton (H^+^) is eliminated from the hydrated water. Thus, the elimination of protons decreased the pH value of the solution through an increase in the H^+^ concentration^30^. This mechanism was represented as follows:

9 $${[{\rm{F}}{\rm{e}}}^{{\rm{I}}{\rm{I}}}{\textstyle \,\text{-}\,}{{\rm{C}}{\rm{N}}{\textstyle \text{-}}{\rm{F}}{\rm{e}}}^{{\rm{I}}{\rm{I}}{\rm{I}}}{\textstyle \,\text{-}\,}{{\rm{O}}{\rm{H}}}_{2}]{\textstyle \,\text{-}\,}{{\rm{C}}{\rm{s}}}^{+}{\to [{\rm{F}}{\rm{e}}}^{{\rm{I}}{\rm{I}}}{\textstyle \,\text{-}\,}{{\rm{C}}{\rm{N}}{\textstyle \text{-}}{\rm{F}}{\rm{e}}}^{{\rm{I}}{\rm{I}}{\rm{I}}}{\textstyle \,\text{-}\,}{{\rm{O}}{\rm{H}}}^{-}]{\textstyle \text{-}}{{\rm{C}}{\rm{s}}}^{+}+{{\rm{H}}}^{+}$$

where [Fe^II^–CN–Fe^III^–OH_2_] is a unit of PB (Figure S1A). During the forward reaction, the increased H^+^ concentration inhibits the adsorption of Cs^+^ onto the composite. Thus, the composite exhibits a lower equilibrium adsorption value at lower pH conditions. The pH variations of DW and SIF during Cs^+^ adsorption over 8 h at different initial Cs^+^ concentrations are shown in Figure S5A. In DW, 1.4-, 2.4- and 5.0-fold increases in pH were observed at the initial Cs^+^ concentrations of 0.1, 1, and 5 ppm, respectively. In contrast, only a small increase in pH was observed in SIF, which may be attributable to the presence of KH_2_PO_4_. As the concentration of KH_2_PO_4_ in SIF is 50 mM, the H_2_PO_4_^−^ ions buffer the SIF solution and stabilize the pH between 7 and 8 (the optimum pH range for Cs^+^ adsorption) at equilibrium^17^ (Figure S5B). From these results, it was clear that the SIF did not limit the equilibrium adsorption of Cs^+^ and the PB-CA composite showed higher adsorption capacity at pH 7–8. In the experiments with 1 ppm Cs^+^ solution, the PB-CA composite showed 99.71%, 88.30%, and 99.43% Cs^+^ removal within 10 min in DW, SGF, and SIF, respectively (Fig. 7). These results indicated that the composite was an efficient adsorbent for the removal of Cs^+^ from the GI tract. Moreover, the selective adsorption of Cs^+^ by the PB-CA composite is evident from the kinetic analysis in SIF, which mimics the physiological conditions of the GI tract with K^+^. In SIF, the composite exhibited the highest Cs^+^ absorption (5.089 mg/g for 5 ppm Cs^+^; Table S1). Therefore, our material selectively adsorbed Cs^+^ without causing any damage to the electrolyte balance of the body.

### Intraparticle diffusion model

**Figure 8:**
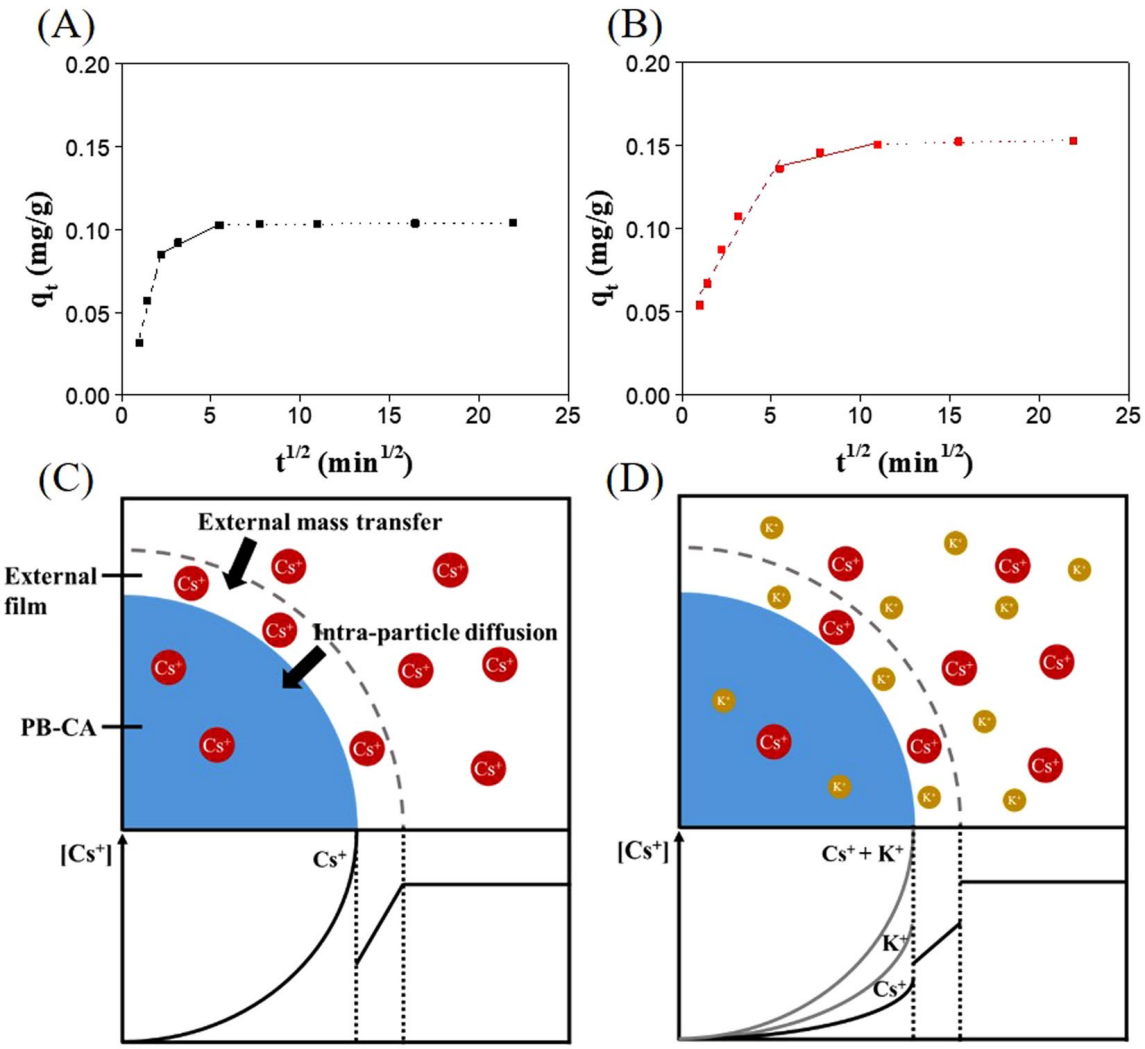
Intra-particle diffusion model of cesium adsorption. (**A**) Diffusion studies of cesium into PB-CA from DW. (**B**) Diffusion studies of cesium into PB-CA from SIF (dashed lines represent external mass transfer, solid lines represent intra-particle diffusion, and dotted lines represent saturation of cesium ions in PB-CA). (**C**) Schematic diagrams of cesium adsorption into the PB-CA from DW. (**D**) Schematic diagrams of cesium adsorption into the PB-CA from SIF (cesium and potassium concentrations are 0.1 and 2000 ppm, respectively).

The pseudo-second-order model reported that Cs^+^ ions were chemisorbed onto the PB-CA composite based on the assumption that chemisorption may be the rate-limiting step. It is most likely that the adsorbate species are transported from the bulk of the solution to the adsorbents through intraparticle diffusion, which is often a rate-limiting step in many adsorption processes^58^. The mechanism of Cs^+^ adsorption into the composite was investigated by an intraparticle diffusion model using the following equation^59^:

10 $${q}_{t}={k}_{p}{t}^{\frac{1}{2}}$$

where the *q*_*t*_ is the adsorption capacity at time *t* and *k*_*p*_ is intra-particle diffusion rate constant (mg/g·min^1/2^). The amount of adsorbed Cs^+^ versus the square root of time (*q*_*t*_ vs *t*^1/2^) (Fig. 8A and B) showed multi-linearity. The plot was divided into three linear regions^60,61^ attributed to: (i) the external mass transfer across the external surface of the adsorbent (the external mass transfer rate constant is *k*_*f*_) or the boundary layer diffusion of solute molecules; (ii) the gradual adsorption stage, where the intraparticle diffusion of the adsorbate onto the adsorbent active site takes place, which is rate-limiting; and (iii) the final equilibrium stage, in which the adsorption sites are saturated and intraparticle diffusion tends to slow down owing to the extremely low concentration of adsorbate in the solution.

**Figure 9:**
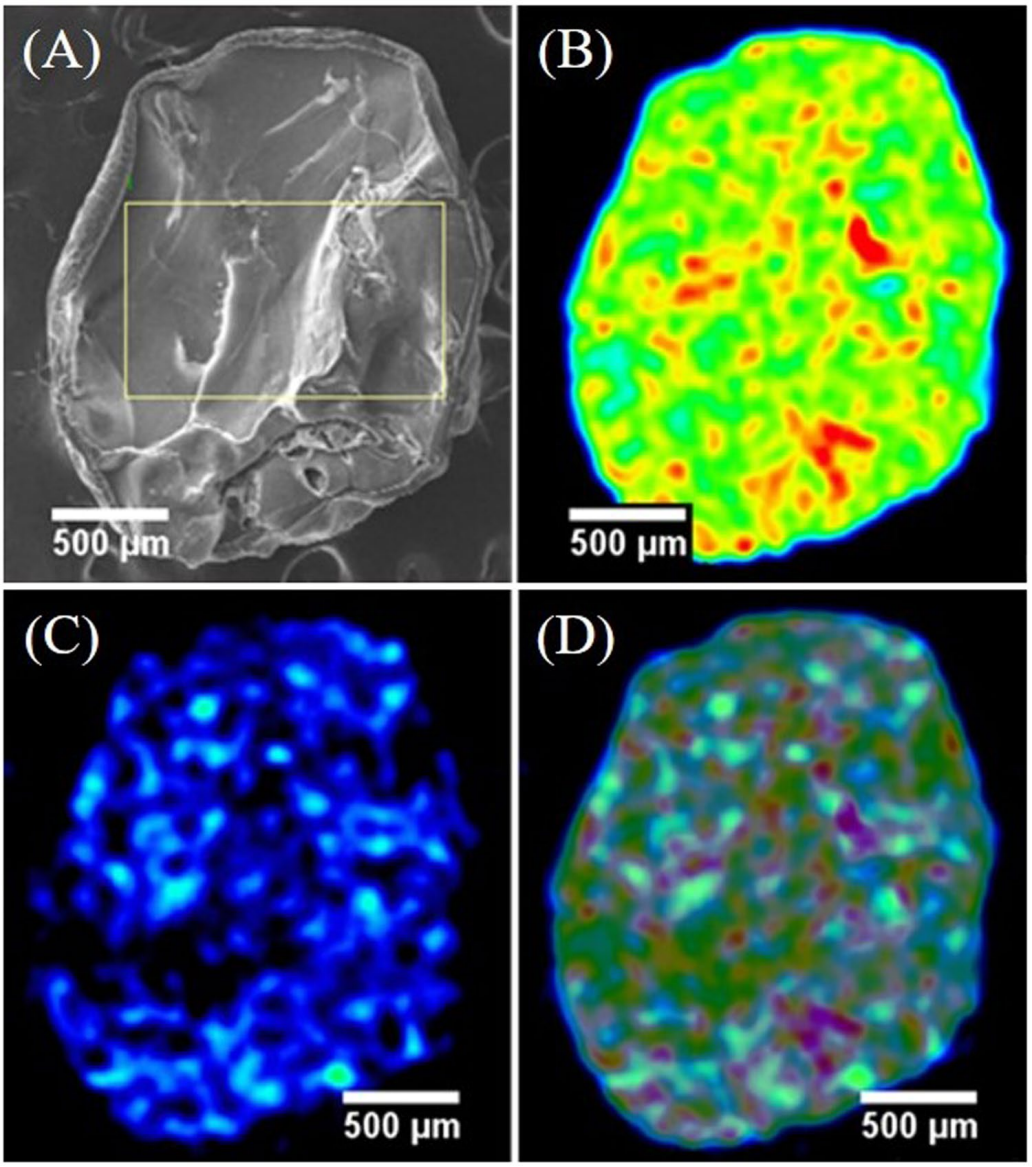
Cesium distribution on PB-CA after adsorption. (**A**) SEM image of the cryo-fractured PB-CA. (**B**) EDS mapping of iron. (**C**) EDS mapping of cesium. (**D**) EDS mapping of iron-cesium-overlay.

Additionally, Fig. 8A and B show that the three processes controlled the rate of Cs^+^ adsorption, but that only one process was rate determining in any particular time interval. The slope of each region of the lines and their rate constant values revealed the adsorption rate. The slope of the external mass transfer (*k*_f_) section was steeper than that of the intraparticle diffusion section (*k*_*p*_), i.e. *k*_*f*_ > *k*_*p*_, in both DW and SIF (Table S2), which indicated that the external mass transfer rate was faster than the intraparticle diffusion step. In turn, this suggested that intraparticle diffusion was a rate-controlling process. The lower slope of the line or the smaller value of the rate constant indicated a slower reaction rate^21^. The values of *k*_*f*_ were 0.0414 and 0.0180 mg g^−1^ min^−1/2^ and the values of *k*_*p*_ were 0.00537 and 0.00256 mg g^−1^ min^−1/2^ in DW and SIF, respectively. DW produced higher *k*_*f*_ and *k*_*p*_ values than SIF, which may be the result of the presence of a significant amount of K^+^ in SIF that hinders the adsorption of Cs^+^. The mole fraction of Cs^+^ to K^+^ was 0.1:2000 ppm. In Fig. 8C and D, it is shown that both Cs^+^ and K^+^ were present in SIF, whereas only Cs^+^ was present in DW. The Cs^+^ concentration gradient between the external film and the intraparticle environment was higher in DW than SIF. The external mass transfer rate and intraparticle diffusion rate of DW were twice that of SIF. The elemental mapping of the composite after the adsorption of 0.1 ppm Cs^+^ in DW for 8 h is shown in Fig. 9. All possible elemental information of Fe, Cs, and the Cs-Fe-overlay are shown to elucidate the distribution of PB and Cs in the PB-CA composite. A cryo-fractured PB-CA composite is shown in Fig. 9A, in which the iron mapping (Fig. 9B) revealed the uniform distribution of PB in the composite. Cs mapping (Fig. 9C) showed the homogeneous dispersion of Cs^+^ onto the composite through the intraparticle diffusion process. The overlaid image of iron with Cs (Fig. 9D) proved the correlation between PB and the Cs^+^ distribution. The mapping data indicated that the Cs^+^ were completely diffused onto the composite, which is a prerequisite for a good adsorbent material.

## Conclusions

An edible PB-CA composite was fabricated as a decorporation agent for the removal of Cs^+^ from the GI tract. The SEM images revealed a 3D porous morphology in which the cubic PB NPs were uniformly distributed on the cellulose matrix. The cell viability analysis confirmed that the composite was not cytotoxic to either THP-1 or Caco-2 cells at the dosages tested. Owing to its larger size than PB, PB-CA was not endocytosed into THP-1 or Caco-2 cells and resulted in a low level of ROS production; thus, the composite was non-cytotoxic. The adsorption studies revealed that the adsorption isotherm was in good agreement with the Langmuir isotherm model and indicated monolayer adsorption with maximum Cs^+^ adsorption capacities of 15.38 and 13.70 mg/g in DW and SIF, respectively. In addition, the composite showed an excellent Cs^+^ removal efficiency of 99.43% in SIF over a period of 10 min. The PB-CA composite showed good stability in both SIF and gamma ray irradiation, which suggested the material was safe for use as an oral treatment agent for Cs^+^ decorporation from GI tract. The Cs^+^ ions were shown to be chemisorbed with an adsorption rate controlled by intraparticle diffusion. The results showed that the composite was an effective adsorbent for Cs^+^ removal in comparison with pure PB with respect to the stability, cell viability, biocompatibility, and adsorption capacity. These findings demonstrated the great potential of the composite for use as an orally administered drug for the decorporation of Cs^+^ from the GI tract.

## Methods

### Materials

Cellulose, pepsin, dimethyl sulfoxide (DMSO), Prussian blue, tetrabutylammonium acetate (TBAA), monopotassium phosphate (KH_2_PO_4_), pancreatin, nickel oxide, L-glutamine, penicillin and streptomycin, phorbol myristate acetate (PMA), Dulbecco’s Modified Eagle’s Medium (DMEM), fetal bovine serum (FBS), Dulbecco’s phosphate-buffered saline (DPBS), and bovine serum albumin (BSA) were purchased from Sigma-Aldrich. 2′, 7′-Dichlorofluorescein diacetate (DCFH-DA) was obtained from Calbiochem, La Jolla, CA, USA. All other reagents were of analytical grade and used as received.

### Synthesis of the PB-CA composite

Briefly, a 2% cellulose solution was prepared by the dissolution of 0.408 g cellulose in a TBAA/DMSO mixture (3 g TBAA/17 g DMSO). The cellulose hydrogel was prepared by the addition of the cellulose solution dropwise to DW. Finally, the as-prepared hydrogel was washed with DW and freeze-dried to obtain the cellulose aerogel (CA). In a typical experiment to prepare the PB-CA composite, approximately 1 mL PB (1 M) solution was added to a 2% cellulose solution and then mixed. The prepared solution was added dropwise to DW to fabricate the PB-cellulose hydrogel. The obtained product was washed with DW and freeze-dried to obtain the PB-CA composite.

### Characterization

X-ray diffraction (XRD) was conducted on a D2 PHASER (Bruker, Germany) and patterns were obtained using CuKα radiation. Fourier transform infrared (FTIR) spectroscopy was conducted by using a Jasco FT/IR-6600. The Brunauer-Emmett-Teller (BET) surface area analysis was conducted on a TriStar II (Micromeritics, GA, USA) was obtained from the N_2_ adsorption/desorption isotherm using a fully automatic physisorption analyzer. Scanning electron microscopy (SEM) was conducted on a Nova NanoSEM 450 (FEI, OR, USA) at an acceleration voltage of 5 kV. SEM-energy dispersive spectroscopy (EDS) mapping was performed using a QuantaX200 (Bruker). X-ray photoelectron spectroscopy (XPS, Thermo Scientific, K-Alpha) was conducted using an Al X-ray source. The UV-vis analysis was performed on a V770 spectrophotometer (JASCO). The pH of the solution was measured by using a JENWAY 3510 pH Meter.

### Calculation of PB concentration in the PB-CA composite

In order to calculate the amount of PB present in the PB-CA composite, approximately 10 mg of PB-CA composite was dispersed in 30 mL of colorless TBAA/DMSO solution. After 12 h, the color of the TBAA/DMSO solution had changed to blue, which indicated the complete dissolution of PB. To calculate the amount of PB in the PB-CA composite, the UV-visible absorbance (Figure S6A) of five different concentrations of the composite were measured and a calibration curve (Figure S6B) was generated. The amount of PB was calculated from the following formula:

11 $${A}_{690}=0.0225\times 5\times [PB]$$

where A_690_ = 0.6376. The amount of PB was calculated to be 1.3045 mg in 10 mg of the PB-CA composite.

### Cell culture

Caco-2 cells obtained from American Type Culture Collection (Manassas, VA, USA) were cultured in DMEM supplemented with 10% FBS, 2 mM L-glutamine, 100 IU/mL penicillin, and 100 U/mL streptomycin. THP-1 cells (American Type Culture Collection, Manassas, VA, USA) were cultured in Roswell Park Memorial Institute 1640 (RPMI-1640) medium supplemented with 10% FBS, 2 mM L-glutamine, 100 IU/mL penicillin, and 100 U/mL streptomycin. Because the THP-1 cells are monocytic, PMA was used to differentiate the macrophage-like cells, as described previously^62^. Both cell types were cultured at 37 °C in an atmosphere of 5% CO_2_.

### Cell viability assay

To evaluate the cell viability, Caco-2 cells were seeded into 96-well plates at a density of 2 × 10^5^ cells/mL and incubated overnight to reach approximately 80% confluence. For differentiated THP-1 cells, monocytic THP-1 cells were seeded at 5 × 10^5^ cells/mL in a 96-well plate and differentiated to macrophages by incubation with 10 ng/mL PMA for 2 days. Both cell types were washed three times with pre-warmed DPBS, followed by the addition of fresh medium containing PB or the PB-CA composite, and incubated for 24 h at doses in the range from 0–1250 μg/mL. After 24 h, the cell viability was measured by using a Cell Counting Kit-8 (CCK-8; Dojindo Molecular Technologies, Gaithersburg, MD, USA). To exclude colorimetric interference from PB or the PB-CA composite, the cells were washed three times with pre-warmed DPBS, the supernatant was removed, and the cells were centrifuged at 15000 × *g* for 10 min. The absorbance was read at 450 nm on a Synergy HT Multi-mode Microplate Reader (Bio-Tek Instruments, Winooski, VT, USA).

### Evaluation of cellular uptake of PB or PB-CA composite

The direct or indirect interactions of chemicals can cause toxicity to cells. To examine the direct mechanism of toxicity, this study evaluated the cellular uptake of the PB-CA composite. Briefly, both cell types were cultured in chamber slides (Lab-Tek, Campbell, CA, USA) using the same protocol as for the cell viability assay. The cells were treated with a sublethal dose of PB (50 μg/mL) or the PB-CA composite (400 μg/mL) for 4 h. The cells were then washed three times with DPBS and fixed with methanol. To visualize the cellular uptake, the cells were stained lightly with eosin, which provided contrast from the blue-stained PB. The intracellular uptake of PB or the PB-CA composite by both cell types was visualized by optical microscopy (Nikon, Tokyo, Japan). The stability of PB or the PB-CA composite was evaluated at 0 and 12 h after incubation with DW, PBS, and DMEM supplemented with 10% FBS or RPMI-1640 supplemented with 10% FBS, because the cellular uptake can be reduced by agglomeration of the particles. The images were captured using a digital camera (Olympus, Korea).

### Cell-free reactive oxygen species (ROS) assay

The intrinsic capability of the overall ROS generation was measured using DCFH-DA by a previously described method^63^. The doses of PB or the PB-CA composite for DCFH-DA assay were 0–1.25 mg/mL and the fluorescence intensities were read at 485/590 nm on a Synergy HT Multi-mode Microplate Reader (Bio-Tek Instruments). The levels of ROS generation were expressed as μM H_2_O_2_ equivalent using a standard curve. Nickel oxide NPs (size: 5.3 ± 1.9 nm; zeta potential: + 48.9 ± 0.6 mV) at 100 μg/mL were used as a positive control^22^.

### Serum protein binding affinity assay

The serum protein binding of PB or the PB-CA composite was evaluated because protein corona formation on the surface of NPs can mitigate cytotoxicity^64^. Briefly, PB or the PB-CA composite was dissolved in DPBS at concentrations between 0 and 1250 μg/mL and incubated with 100 μg/mL BSA for 4 h at room temperature. PB or the PB-CA composite was then removed by centrifugation at 15000 × *g* for 10 min and the levels of protein in the supernatant were measured by using the bicinchoninic acid (BCA) assay (Thermo Fischer Scientific, Rockford, IL, USA).

### Preparation of the SGF and SIF

To prepare SGF, 3.2 g pepsin was dissolved in acidic aqueous solution (7 mL of 37 wt% HCl in 1 L DW). To prepare SIF, 6.505 g KH_2_PO_4_ first was dissolved in 1 L DW to produce 0.05 M KH_2_PO_4_. Subsequently, 1 M NaOH was added to maintain the pH at 7.5 and then mixed with 1 g pancreatin.

### Stability of PB-CA composite

We investigated the stability of the PB-CA composite in the digestive fluid and under gamma-ray irradiation from a ^60^Co source. In a typical experiment, 10 mg of the composite (containing 1.3045 mg PB NPs) was dispersed in 20 mL SGF or SIF for 24 h. The release of PB from the composite was measured by UV-vis spectrophotometric analysis. Similar experiments were performed to compare the pure PB NPs with the PB-CA composite. In contrast, for gamma irradiation experiments, the aqueous dispersion of the composite was irradiated at doses of 0, 6, and 60 kGy. After irradiation, the solutions were analyzed by UV-vis spectroscopy. The irradiation facility was provided by Korea Atomic Energy Research Institute (KAERI), Republic of Korea.

### Adsorption isotherm

Cesium adsorption studies using the PB-CA composite were performed in both DW and SIF. The adsorption isotherms were performed based on batch experiments using inactive Cs, in which the initial concentration of Cs^+^ was between 1 and 500 ppm. A fixed amount of adsorbent (10 mg) was added to 10 mL of an aqueous Cs^+^ solution and shaken at 40 rpm on a rotary shaker for 24 h. After equilibrium was reached, the adsorbent was separated by filtration and the Cs^+^ concentration was analyzed by inductively coupled plasma-mass spectrometry (ICP-MS).

### Conversion of Radioactive to Non-radioactive Cs Concentration

In order to study adsorption kinetics, non-radioactive Cs concentrations (0.1, 1, and 5 ppm) were determined by the stagnant water Cs concentration. The maximum ^137^Cs concentration in the Fukushima-1 nuclear power plant is 3.0 × 10^6^ Bq/mL (3.0 × 10^9^ Bq/L)^65^. As 1 g of ^137^Cs has an activity of 88 Ci/g (3.26 × 10^9^ Bq/mg)^66^, the Cs concentration was calculated as follows:

12 $$(3.0\times {10}^{9}\frac{Bq}{L})\,\times \,(\frac{1}{3.26\times {10}^{9}}\frac{mg}{Bq})\cong 0.92\,ppm$$

The maximum Cs concentration was calculated to be 0.92 ppm.

### Adsorption kinetics

The adsorption kinetics was determined by dispersing 10 mg of PB-CA each in 0.1, 1 and 5 ppm inactive cesium solution (20 ml) in a rotary shaker operated at 40 rpm. The samples were collected at different times (1, 2, 5, 10, 30, 60, 120, 240, 270, and 480 min).

## Electronic supplementary material

Supplementary Information
