# Supplementary material for: Porous 3D Prussian blue/cellulose aerogel as a decorporation agent for removal of ingested cesium from the gastrointestinal tract
Source: Sci Rep. 2018 Mar 14;8:4540. doi: 10.1038/s41598-018-22715-w (PMC5851989; doi:10.1038/s41598-018-22715-w)
Supplement: Supplementary file 1 — Supplementary Information [file 41598_2018_22715_MOESM1_ESM.docx]

Porous 3D Prussian blue/cellulose aerogel as a decorporation agent for removal of ingested cesium from the gastrointestinal tract

Ilsong Lee^1,2,+^, Sung-Hyun Kim^3,+^, Muruganantham Rethinasabapathy^1^, Yuvaraj Haldorai^4^, Go-Woon Lee^1,5^, Sang Rak Choe^1^, Sung-Chan Jang^1,2^, Sung-Min Kang^1^, Young-Kyu Han^4^, Changhyun Roh^2,6,^*, Wan-Seob Cho^3,^*, and Yun Suk Huh^1,^*

^1^ Department of Biological Engineering, Biohybrid Systems Research Center (BSRC), Inha University, 100, Inha-ro, Incheon 22212, Republic of Korea
^2^ Biotechnology Research Division, Advanced Radiation Technology Institute (ARTI), Korea Atomic Energy Research Institute (KAERI), 29, Geumgu-gil, Jeongeup-si, Jeonbuk 56212, Republic of Korea
^3^ Laboratory of Toxicology, Department of Medicinal Biotechnology, College of Health Sciences, Dong-A University, 37, Nakdong-daero, 550 beon-gil, Busan 49315, Republic of Korea
^4^ Department of Energy and Materials Engineering, Dongguk University-Seoul, 30 Pildong-ro 1-gil, Seoul 04620, Republic of Korea
^5^ R&D Platform Center, Korea Institute of Energy Research (KIER), 152, Gajeong-ro, Deajeon 34129, Republic of Korea
^6^ Radiation Biotechnology and Applied Radioisotope Science, University of Science and Technology (UST), 217, Gajeong-ro, Daejeon 34113, Republic of Korea

* Corresponding Authors

(C.R.) chroh@kaeri.re.kr; (W.-S.C) wcho@dau.ac.kr; (Y.S.H) yunsuk.huh@inha.ac.kr

^+^ These authors contributed equally to this work.


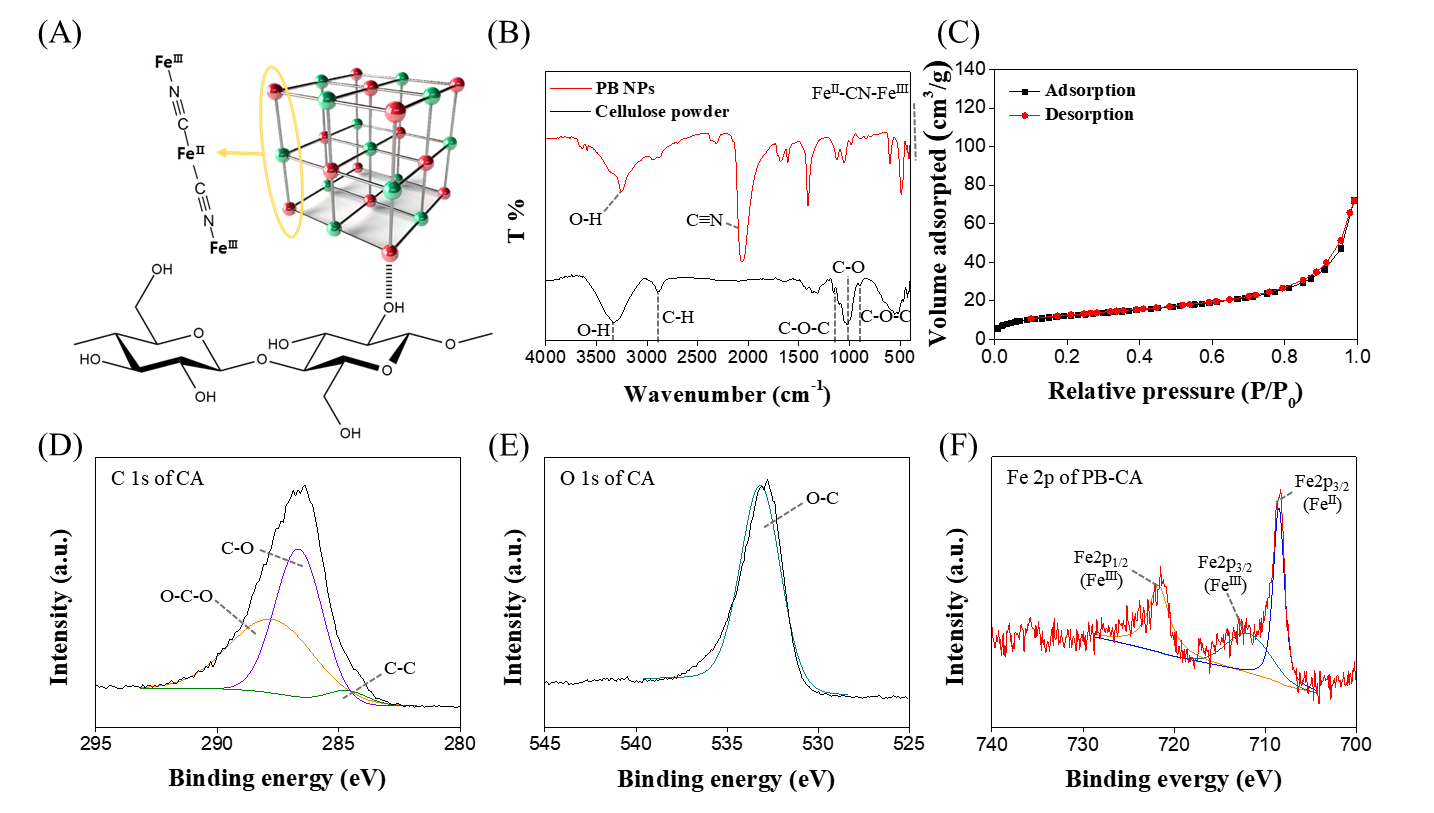


**Figure S1**. Characterization of PB-CA. (A) Schematic diagram of the interaction of cellulose with PB NP. (B) FT-IR spectra of PB NPs and cellulose powder. (C) The BJH N2 adsorption/desorption isotherms of CA. (D) C1 spectrum of PB-CA. (E) C1 spectrum of CA. (F) Fe2p spectrum of PB-CA.


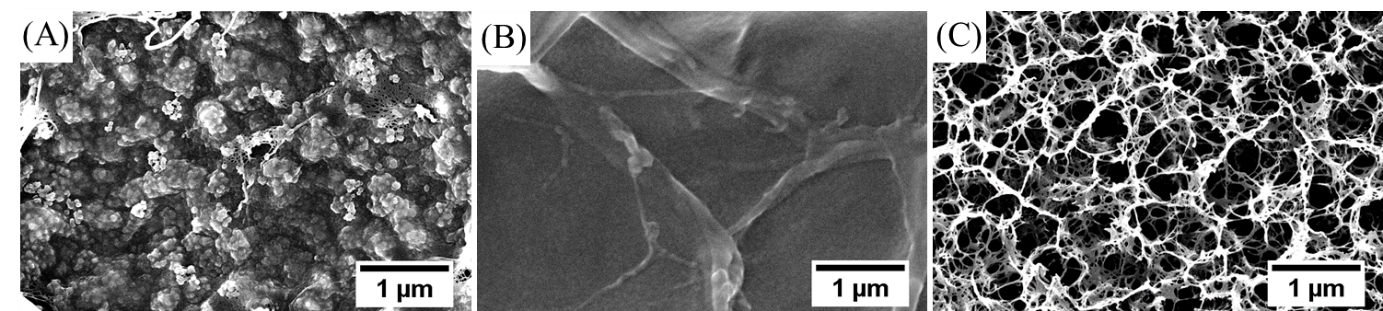


**Figure S2**. SEM images of PB-CA and CA. (A) Surface morphology of PB-CA. (B) Surface morphology of CA. (C) Inner structure of CA.


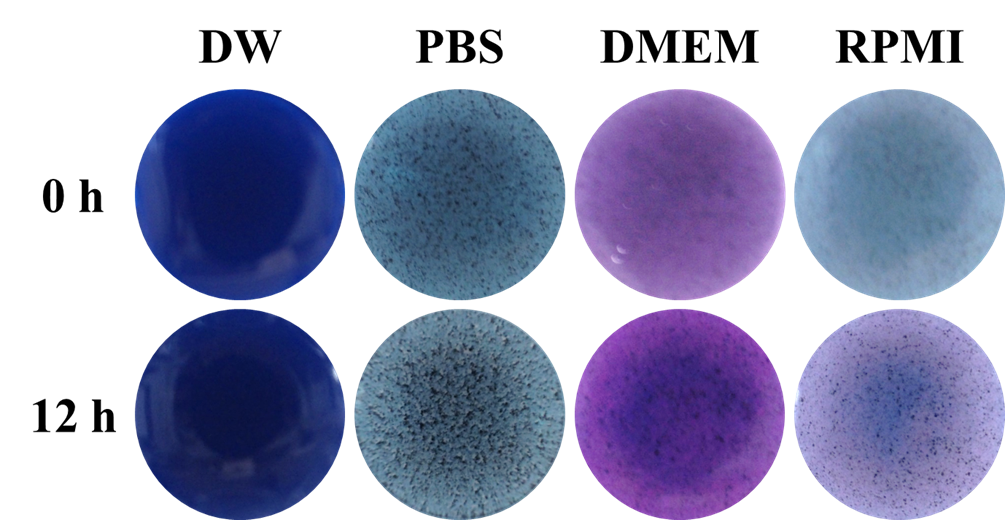


**Figure S3**. The agglomeration status of PB in various media at 0 h (immediately after dispersion) and 12 h after dispersion.


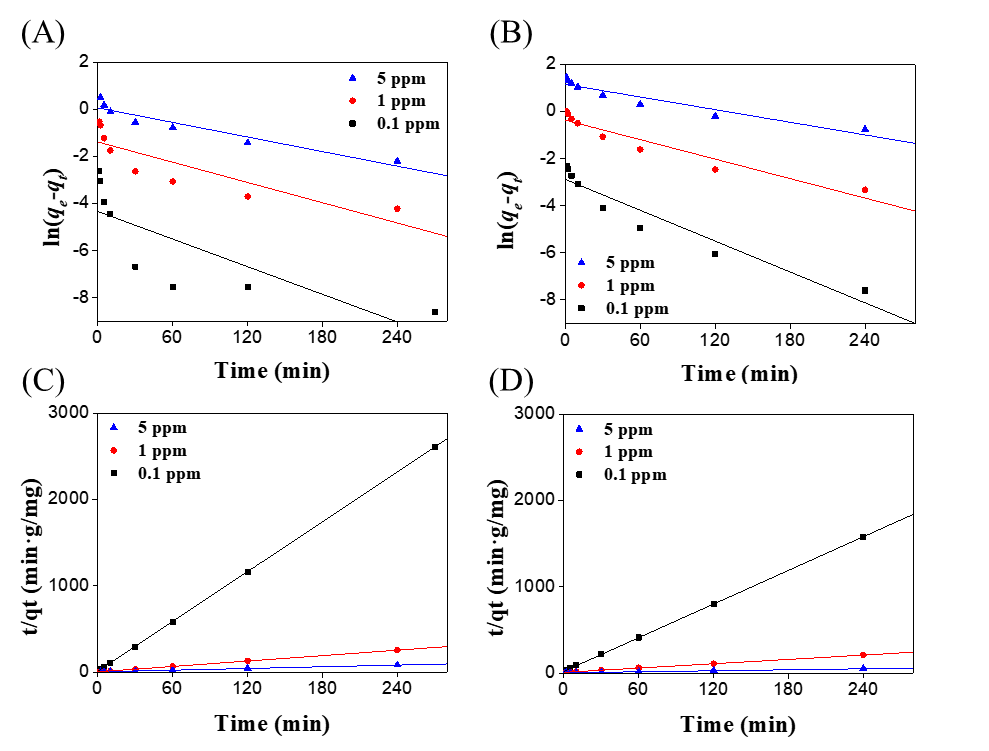


**Figure S4**. (A) Pseudo-first-order cesium adsorption kinetics of PB-CA in DW. (B) Pseudo-first order kinetics of PB-CA in SIF. (C) Pseudo-second-order cesium adsorption kinetics of PB-CA in DW. (D) Pseudo-second-order cesium adsorption kinetics of PB-CA in SIF. The concentrations of cesium ions are 0.1, 1 and 5 ppm.

**Table S1.** Cesium adsorption kinetics parameters of PB-CA in DW and SIF.

|  | | DW | | | SIF | | |
| --- | --- | --- | --- | --- | --- | --- | --- |
|  |  | 0.1 ppm | 1 ppm | *5* ppm | 0.1 ppm | 1 ppm | 5 ppm |
| Pseudo first | *k_1_*  [min^-1^] | 0.020 | 0.014 | 0.010 | 0.021 | 0.014 | 0.001 |
|  | *q_e_*  [mg/g] | 0.013 | 0.248 | 1.058 | 0.056 | 0.694 | 3.170 |
|  | *R^2^* | 0.5634 | 0.7021 | 0.8876 | 0.9050 | 0.9059 | 0.8916 |
| Pseudo second | *k_2_*  [g/mg min] | 7.893 | 0.400 | 0.050 | 2.027 | 0.084 | 0.013 |
|  | *q_e_*  [mg/g] | 0.104 | 0.957 | 3.044 | 0.154 | 1.200 | 5.089 |
|  | *R^2^* | 0.9994 | 0.9999 | 0.9999 | 0.9964 | 0.9997 | 0.9999 |


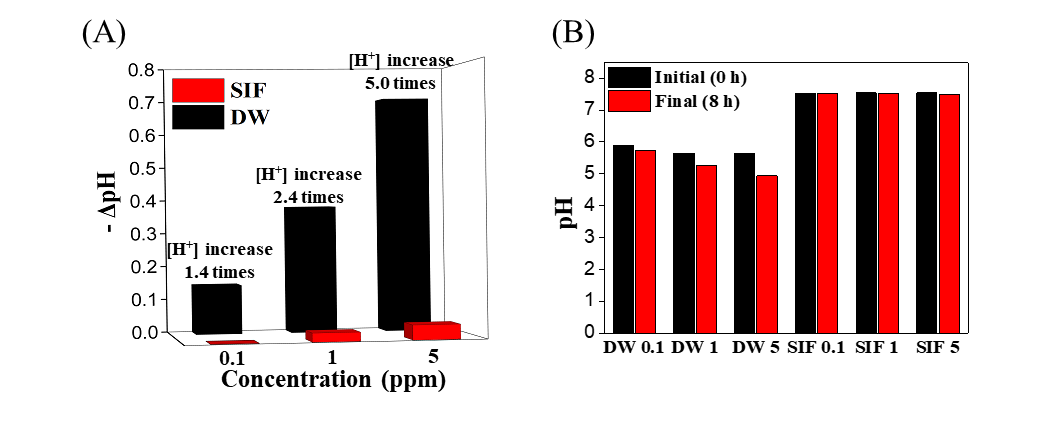


**Figure S5**. (A) Change in pH observed after cesium adsorption of PB-CA in DW and SIF for 8 h. (B) pH values observed after cesium adsorption of PB-CA in DW and SIF for 8 h. The initial concentrations of cesium ions are 0.1, 1, and 5 ppm.

**Table S2.** Intra-particle diffusion rate constant of cesium adsorption into PB-CA from DW and SIF. Cesium concentration is 0.1 ppm.

|  | DW | SIF |
| --- | --- | --- |
| *k_f_*  [mg/g min^1/2^] | 0.0414 | 0.0180 |
| *R^2^* | 0.9471 | 0.9520 |
| *k_p_*  [mg/g min^1/2^] | 0.00537 | 0.00256 |
| *R^2^* | 0.9565 | 0.8400 |


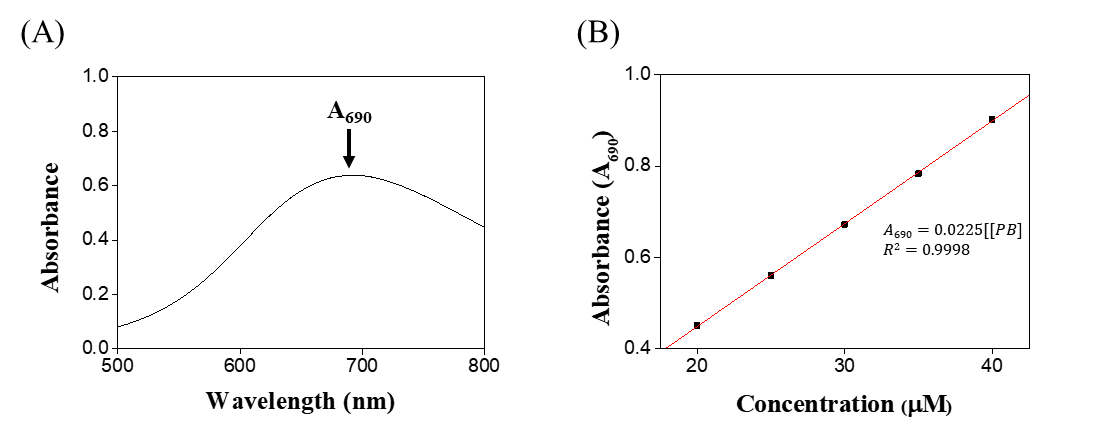


**Figure S6**. (A) UV-vis of 10 mg PB-CA dissolved in 30 ml TBAA/DMSO solution. (B) Calibration curve of PB dissolved in TBAA/DMSO solution.
